# Complete Genome Sequence of a Hobi-Like Virus Isolated from a Nelore Cow with Gastroenteric Disease in the State of São Paulo, Brazil

**DOI:** 10.1128/genomeA.00767-17

**Published:** 2017-08-17

**Authors:** Adriana Cortez, João Pessoa Araújo, Eduardo Furtado Flores, Márcio Garcia Ribeiro, Jane Megid, Antonio Carlos Paes, José Paes de Oliveira Filho, Leila Sabrina Ullmann, Camila Dantas Malossi, Marcos Bryan Heinemann

**Affiliations:** a Universidade Santo Amaro –UNISA, Curso de Medicina Veterinária, São Paulo, São Paulo, Brazil; b Universidade Estadual Paulista “Júlio de Mesquita Filho”–UNESP, Instituto de Biociências, Botucatu, São Paulo, Brazil; c Departamento de Medicina Veterinária Preventiva, Universidade Federal de Santa Maria, UFSM, Santa Maria, Rio Grande do Sul, Brazil; d Faculdade de Medicina Veterinária e Zootecnia, Universidade Estadual Paulista “Júlio de Mesquita Filho”–UNESP, Botucatu, São Paulo, Brazil; e Faculdade de Medicina Veterinária e Zootecnia, Universidade de São Paulo–USP, São Paulo, São Paulo, Brazil

## Abstract

The Hobi-like virus presents antigenic and molecular differences in relation to bovine virus diarrhea virus 1 and 2. The description of the complete genome of the Hobi-like virus SV757/15, isolated from a Nelore cow with gastroenteric disease in Brazil, will help in understanding the evolution and diversity of pestiviruses.

## GENOME ANNOUNCEMENT

The Hobi-like virus BVDV-3, an atypical pestivirus, was first identified in bovine fetal serum from Brazil (1). Although it presents clinical similarity to bovine virus diarrhea virus 1 (BVDV-1) and 2 (BVDV-2) in hosts, it has antigenic and genomic differences that may interfere with diagnosis and protection by vaccination (2–5).

The strain SV757/15 was isolated from intestinal fragments from a 32-month-old Nelore cow that presented severe lesions, in Torre de Pedra, São Paulo, Brazil. The virus isolate (infected MDBK cell culture supernatant) was centrifuged at 14,000 × *g* for 10 min at 4°C. It was then filtered with 0.45-µm disk filters (Millipore), followed by nuclease treatment (50 U of Ambion Turbo DNase in 150 µL with incubation at 37°C for 1 h) and RNA purification using a total RNA purification kit (Norgen Biotek). The RNA concentration was determined using a Qubit fluorimeter (Invitrogen). cDNA was synthesized using the RevertAid first-strand cDNA synthesis kit (Thermo Fisher Scientific) and random hexamer primers, followed by double-strand cDNA synthesis using RNase H, T4 DNA polymerase, and T4 DNA ligase (Thermo Scientific), as previously described (6). Nextera XT libraries (Illumina) were prepared using 1 ng of double-stranded cDNA, quantified using a Kapa library quantification kit for Illumina platforms (Kapa Biosystems) diluted to 1 nM, and sequenced on the NextSeq system (Illumina) using a NextSeq 500 mid-output kit (150 cycles).

The initial quality of each sample was assessed using the “QC Report” tool of CLC Genomics Workbench version 9.1. Based on the initial quality reports, filtration was performed using the “Trim Sequences” tool of CLC Genomics Workbench version 9.1. The filtration parameters consisted of removal of low-quality sequences and sequences containing more than 2 ambiguous nucleotides, as well as fixed trimming of 19 nucleotides (nt) from the 5′ end and 5 nt from the 3′ end. Reads that were less than 50 nt in length after filtration were discarded. Initial assembly for all of the sequences was performed using the *de novo* assembly strategy of CLC Genomics Workbench version 9.1. The contigs were used to map to a Hobi-like complete genome sequence that had previously been published (GenBank accession no. JX985409), and the number of predicted polyprotein-coding genes was similar to that of the same reference sequence. The strain SV757/15 comprises 12,252 nt with lengths of 440 nt and 11 nt at the 5′ and 3′ ends, respectively, and one open reading frame encompassing 3,899 amino acids (12,252 nt).

The alignment and identity matrix with the sequences available in GenBank were produced using Bioedit software (http://www.mbio.ncsu.edu/BioEdit/bioedit.html), and the phylogenetic tree was reconstructed using Mega version 6.0 software (7). The SV757/15 genome presented similarities ranging from 90.7% to 96.8% with the Hobi-like sequences and from 65.9% to 66.4% and 64.3% to 66.4% with the BVDV-1 and BVDV-2 sequences, respectively. In the phylogenetic analysis using the entire genome, it was observed that isolate SV757/15 formed a brother clade with samples that were predominantly from Italy (GenBank accession no. KJ627180, JO612705, KJ6271179, JQ612704, KC788748, and HQ231763).

### Accession number(s).

The complete genomic sequence of SV757/15 has been deposited in GenBank under the accession no. KY683847.
